# Tubeless PCNL versus standard PCNL for the treatment of upper urinary tract stones: a propensity score matching analysis

**DOI:** 10.1007/s11255-023-03872-y

**Published:** 2023-11-21

**Authors:** Yunwu Hao, Xudong Shen, Dongbing Han, Zongyao Hao, Degang Chen

**Affiliations:** 1 Department of Urology, Lu’an Hospital Affiliated of Anhui Medical University, Lu’an, 237000 Anhui China; 2 Department of Urology, The First Affiliated Hospital of Anhui Medical University, Hefei, 230022 Anhui China; 3 Institute of Urology, Anhui Medical University, Hefei, 230022 Anhui China; 4 Anhui Province Key Laboratory of Genitourinary Diseases, Anhui Medical University, Hefei, 230022 Anhui China

**Keywords:** Percutaneous nephrolithotomy, Tubeless, Upper urinary tract, Surgery

## Abstract

**Purpose:**

In this study, the feasibility of tubeless percutaneous nephrolithotomy (PCNL) for the treatment of upper urinary tract stones was investigated.

**Methods:**

From January 2021 to December 2022, the clinical data of 273 patients who received tubeless PCNL (Group A) were studied. The control group includes clinical data from 302 patients (from January 2019 to October 2022) who received standard PCNL (Group B). The baseline characteristics were consistent between the two groups after using the propensity score matching (PSM) method. Compare the preoperative clinical characteristics, postoperative complications, residual stones, catheterization time, and hospital stay between the two groups.

**Results:**

146 pairs of patients were successfully paired through PSM. There was no statistically significant difference in operative time, blood leukocyte counts, haemoglobin decrease, fever, urinary extravasation, sepsis, bleeding, blood transfusion rates, embolism, and residual stones after surgery between the two groups; Postoperative day 1 and discharge day, the VAS pain score in Group A was significantly lower than that in Group B. The catheterization time and hospitalization time of patients in Group A were significantly lower than those in Group B.

**Conclusion:**

According to the inclusion and exclusion criteria, selecting suitable patients for tubeless PCNL is safe and effective, while significantly alleviating pain and reducing catheterization time and hospital stay.

## Introduction

Upper urinary tract stone is one of the most common diseases in Urology. Its incidence rate is increasing year by year worldwide [1], of which about 70% of kidney stone disease may recur after surgery [2]. When stones cause upper urinary tract obstruction, it can lead to hydronephrosis, urinary system infection, renal failure, sepsis and even life-threatening [3]. Percutaneous nephrolithotomy (PCNL) has become a first-line treatment for kidney stone with a diameter greater than 2 cm [4].

It is usually necessary to indwelling a nephrostomy tube after PCNL because it has the function of draining urine, preventing urinary extravasation, and compressing hemostasis [5]. However, there are also relevant reports that indwelling a nephrostomy tube after PCNL can lead to prolonged hospital stay, urinary extravasation, and postoperative pain [6]. The technique of using non indwelling nephrostomy after PCNL to reduce catheter related complications is called tubeless PCNL [7, 8]. This study combined minimally invasive and tubeless aspects of PCNL, and collected the clinical data of tubeless PCNL and standard PCNL patients, and compared them after matching with propensity score matching (PSM). This study found that tubeless PCNL did not increase the incidence of surgical complications, and it is safe and effective for selecting appropriate patients for treatment, which is of great significance for simplifying the PCNL surgical process.

## Materials and methods

### Patients

From January 2021 to December 2022, 273 patients from Urology, Lu’an Hospital affiliated to Anhui Medical University were selected to receive tubeless PCNL. According to the inclusion and exclusion criteria, 158 patients were selected as the study group (Group A). Between January 2019 and October 2022, 302 patients underwent standard PCNL, among which the clinical data of 198 patients with postoperative indwelling nephrostomy after surgery were chosen based on the inclusion and exclusion criteria as the control group (Group B). Inclusion criteria: (1) clear diagnosis after urological ultrasound, CT, KUB + IVP examination; (2) stone diameter > 2 cm; (3) renal cortical thickness > 4 mm; (4) no urinary system infection or urinary system infection has been controlled before surgery. Exclusion criteria: (1) patients with hypertension, diabetes, renal failure and other chronic diseases before surgery, whose blood pressure or blood sugar has not been effectively controlled; Patients with severe illnesses such as dysfunction of important organs such as the heart, brain, and lungs; Patients with coagulation disorders and a history of malignant tumors who cannot tolerate surgery and anesthesia; (2) patients with functional or anatomical solitary kidney, as well as those with stones in the opposite kidney that require surgical treatment; (3) patients with severe lumbar scoliosis, paraplegia, and urinary system without congenital malformation or anatomical structure abnormality, such as: sponge kidney, horseshoe kidney, ectopic kidney, polycystic kidney, calyceal diverticulum with stones, transplanted kidney, ureteral stenosis, and renal pelvis urinary junction stenosis; (4) patients who are found to have severe urinary tract infections during surgery (considering purulent fluid in the kidney or infectious stones) need to terminate the surgery and plan a second phase surgery; (5) patients with high stone load who are unable to completely remove the stones in one stage, as well as patients who require dual or even multi-channel percutaneous nephrolithotomy treatment.

**Fig. 1:**
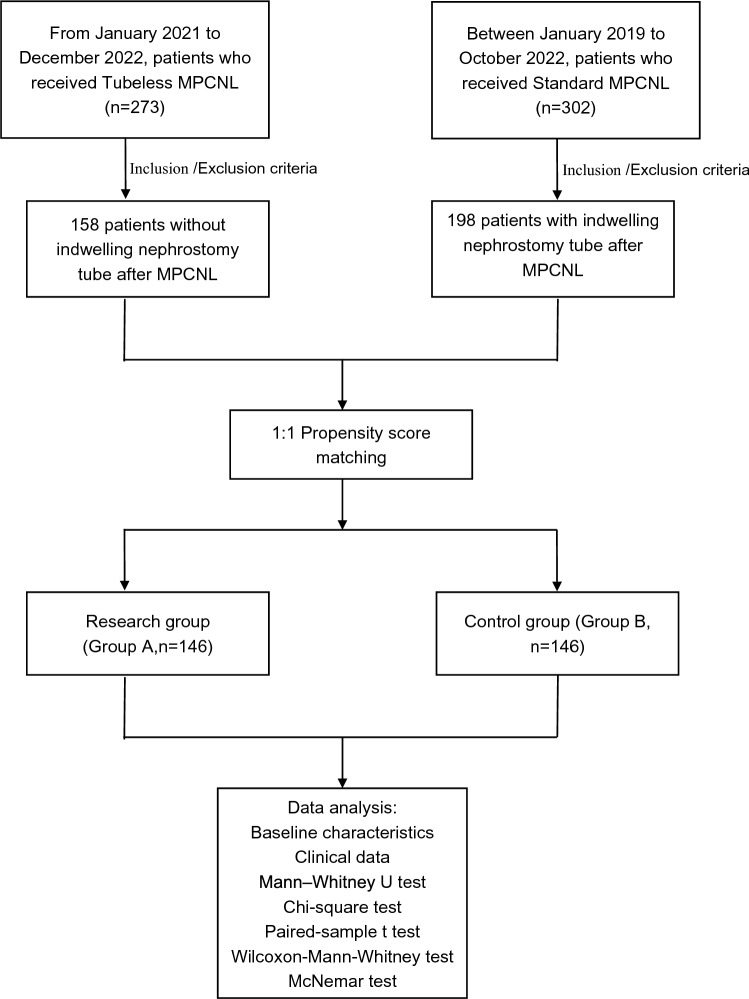
Flowchart of study population

We obtained clinical data from 146 pairs of patients after conducting PSM on potential confounding factors such as gender, age, degree of hydronephrosis, and stone size. The surgical indications are in accordance with guidelines on urolithiasis of the American Association of Urology [9]. The design of the study was approved by the Ethics Committee at Lu’an Hospital Affiliated of Anhui Medical University, and all patients gave written and informed consent to participate. This study was performed in accordance with the Declaration of Helsinki and with standards of good clinical practice. Figure 1 presents a flowchart describing the selection of the study population.

### Equipment and techniques

After successful general anesthesia, the patient takes the lithotomy position. Under the observation of ureteroscope, the F5 Ureteric stent was placed in the calculus side ureter and fixed on the F16 Foley catheter. Change to prone position, inject normal saline through ureteric stent to establish artificial kidney hydronephrosis; under the guidance of ultrasound, an 18G puncture needle is used to puncture the target renal calyx through the 11th intercostal or 12th subcostal region. After urine flows out, a J-shaped guide wire is inserted through the puncture needle, and a fascia dilator is placed along the guide wire to expand the channel. Starting from F8, it gradually expands to F18 or F20, and a working channel is established using F18 or F20 plastic sheath. The ureteroscope was used to enter the renal collection system through the working channel, and a perfusion pump was used for flushing. The stones were removed by lithotripsy using Ricken holmium laser (2.0 J and 40 Hz), and residual stones were examined by ultrasound before the surgery ended. Group A removed the plastic sheath and only retained the double J tube; Group B simultaneously retained double J tubes and nephrostomy tubes. Two groups returned to the hospital 2–4 weeks after surgery to remove the double J tube.

The operation time was defined as from retrograde placement of Ureteric stent to the end of nephrostomy. Sepsis is defined according to the 2001 International Conference on the Definition of Sepsis [10]. Infection occurs within 48 h after surgery and at least two conditions occur: (1) heart rate greater than 90 beats/minute; (2) body temperature over 38 or under 36 degrees Celsius; (3) leukocyte count greater than 12,000 or less than 4000/microliters; (4) respiratory rate greater than 20 breaths/minute. Residual stones refers to the discovery of clinically significant residual fragments with a total diameter greater than 4 mm on postoperative urological X-ray examination. Surgical complications are classified according to the Clavien Dindo grading system [11].

### Observation indicator

Compare the preoperative baseline data, operative time, haemoglobin decrease, postoperative fever, blood leukocyte counts, analgesic requirement, urinary extravasation, sepsis, bleeding, transfusion rate, embolization, urinary extravasation, residual stones, catheterization time, and hospital stay between the two groups of patients.

### Statistical analysis

The statistical analysis was performed using SPSS 25 (IBM Corp, Armonk, NY, USA). PSM was implemented using the PSM extension procedure in IBM SPSS. The nearest neighbour algorithm was used as the matching method, and the calliper value set to 0.02 and the ratio set to 1:1. Before matching, independent sample t tests were used for the data with a normal distribution, and Mann‒Whitney U tests were used for the data with an abnormal distribution. Chi-square tests were used to compare the categorical variables. After matching, normally distributed variable data were analysed with a paired-sample t test. Wilcoxon-Mann‒Whitney tests were used for nonnormally distributed variable data, and categorical variable data were analysed with the McNemar test. *P* < 0.05 was considered statistically significant. All of the experimental data are displayed as the average value ± standard deviation.

## Results

There was a significant difference in stone size between the two groups before matching (Table 1). We performed PSM for a total of 146 pairs that were matched successfully. The ages of patients in Group A and Group B were 53 (21–80) years and 52 (21–79) years, respectively. Preoperative baseline characteristics such as gender, age, body mass index (BMI), symptom, treatment history, past history, degree of hydronephrosis, urinary leucocyte, stone size, urine culture, operation site, and stone location were not statistically significant between the two groups (*P* > 0.05, Table 2). The operative time for Group A and Group B were 79.5 ± 16.2 min and 81.0 ± 14.5 min, respectively; The haemoglobin decrease for Group A and Group B were 10.0 ± 7.7 g/dl and 9.1 ± 6.7 g/dl, with postoperative bleeding in two groups were 12 (8.2%) and 10 (6.8%) cases; Postoperative fever was found in 21 (14.4%) and 23 (15.7%) cases, respectively; blood leukocyte counts in two groups were 9.1 ± 3.0 × 10^9^/L and 9.2 ± 2.9 × 10^9^/L, and postoperative sepsis occurred in 4 (2.7%) cases and 3 (2.1%) cases; urinary extravasation for Group A and Group B were found in 3 (2.1%) cases and 9 (14.4%) cases; transfusion rate in two groups were 3 (2.1%) and 2 (1.4%), with embolization in 6 (4.1%) cases and 2 (1.4%) cases; postoperative residual stones were found in 15 (10.3%) and 12 (8.2%) cases, respectively; the differences were not statistically significant (*P* > 0.05). Analgesic requirement were 19 (13.0%) cases and 30 (20.5%) cases, and VAS pain score of group A was significantly lower than that of group B; the catheterization time for the two groups of patients was 3.3 ± 1.2 days and 4.2 ± 1.1 days, and the hospital stay was 6.8 ± 1.6 days and 7.7 ± 1.6 days, respectively. The difference was statistically significant (*P* < 0.05, Table 3).

**Table 1:** Baseline criteria of the two groups before PSM

	Group A	Group B	*P* value
Patients, *n*	158	198	
Gender^b^			
Male	104 (66%)	129 (65%)	0.895
Female	54 (34%)	69 (35%)	
Age median (min–max)^a^	52 (20–80)	51 (21–81)	0.735
BMI (kg/m^2^)^a^	24.8 ± 2.5	24.6 ± 2.3	0.312
Symptom			
Lumbago^b^	108 (68.3%)	140 (70.7%)	0.631
Symptomless^b^	44 (27.8%)	52 (26.3%)	0.738
Pyrexia^b^	15 (9.5%)	20 (10.1%)	0.848
Vomiting^b^	28 (17.7%)	44 (22.2%)	0.294
Hematuria^b^	18 (11.4%)	21 (10.6%)	0.797
Treatment history			
ESWL^b^	12 (7.6%)	14 (7.1%)	0.850
Ureteroscopic lithotripsy^b^	12 (7.6%)	13 (6.6%)	0.706
Percutaneous nephrolithotomy^b^	17 (10.7%)	17 (8.6%)	0.488
Past history			
Hypertension^b^	44 (27.8%)	54 (27.3%)	0.904
Diabetes^b^	14 (8.8%)	16 (8.1%)	0.792
Degree of hydronephrosis^b^			
No	41 (25.9%)	56 (28.3%)	0.931
Mild	57 (36.1%)	71 (35.8%)	
Moderate	50 (31.6%)	60 (30.3%)	
Severe	10 (6.3%)	11 (5.6%)	
Urinary leucocyte^b^			
No	38 (24.1%)	44 (22.2%)	0.559
Possible	33 (20.9%)	51 (25.8%)	
Yes	87 (55.0%)	103 (52.0%)	
Urinary nitrite^b^	14 (8.8%)	16 (8.1%)	0.792
Urine culture^b^	22 (13.9%)	32 (16.2%)	0.545
*E. coli*	10	14	
Enterococcus	4	5	
*Proteus mirabilis*	2	2	
Other	6	11	
Stone size (mm)^a^	23.6 ± 8.9	25.7 ± 8.4	0.001
Operation site^b^			
Left	82 (51.9%)	100 (50.5%)	0.794
Right	76 (48.1%)	98 (49.5%)	
Stone location			
Ureteric stone^b^	26 (16.5%)	29 (14.6%)	0.621
Renal and ureteric stone^b^	28 (17.7%)	30 (15.2%)	0.986
Renal stone^b^	89 (56.3%)	120 (60.6%)	0.586
Staghorn stone^b^	15 (9.5%)	19 (9.6%)	0.953

^a^Nonnormally distributed variable data analysed with the Mann‒Whitney U test

^b^Categorical variable data analysed with the chi-square test

**Table 2:** Baseline criteria of the two groups after PSM

	Group A	Group B	*P* value
Patients, *n*	146	146	
Gender^b^			
Male	96 (65.8%)	101 (69.2%)	0.625
Female	50 (34.2%)	45 (30.8%)	
Age median (min–max)^d^	53 (21–80)	52 (21–79)	0.895
BMI (kg/m^2^)^c^	24.7 ± 2.5	24.8 ± 2.3	0.875
Symptom			
Lumbago^e^	100 (68.5%)	103 (70.5%)	0.795
Symptomless^e^	40 (27.4%)	37 (25.3%)	0.787
Pyrexia^e^	14 (9.6%)	15 (10.3%)	1.000
Vomiting^e^	28 (19.2%)	27 (18.5%)	1.000
Hematuria^e^	17 (11.6%)	17 (11.6%)	1.000
Treatment history			
ESWL^e^	10 (6.8%)	11 (7.5%)	1.000
Ureteroscopic lithotripsy^e^	11 (7.5%)	11 (7.5%)	1.000
Percutaneous nephrolithotomy^e^	12 (8.2%)	14 (9.6%)	0.824
Past history			
Hypertension^e^	42 (28.8%)	41 (28.1%)	0.889
Diabetes^e^	13 (8.9%)	13 (8.9%)	1.000
Degree of hydronephrosis^e^			
No	39 (26.7%)	40 (27.4%)	0.911
Mild	53 (36.3%)	52 (35.6%)	
Moderate	46 (31.5%)	44 (30.1%)	
Severe	8 (5.48%)	10 (6.8%)	
Urinary leucocyte^e^			
No	37 (25.3%)	33 (22.6%)	0.555
Possible	32 (21.9%)	35 (24.0%)	
Yes	77 (52.8%)	78 (53.4%)	
Urinary nitrite^e^	13 (8.9%)	12 (8.2%)	1.000
Urine culture^e^	21 (14.4%)	23 (15.8%)	0.864
*E. coli*	10	11	
*Enterococcus*	4	3	
*Proteus mirabilis*	2	3	
Other	5	6	
Stone size (mm)^d^	23.9 ± 9.0	24.3 ± 7.1	0.462
Operation site^e^			
Left	74 (50.7%)	77 (52.7%)	0.810
Right	72 (49.3%)	69 (47.3%)	
Stone location			
Ureteric stone^e^	23 (15.8%)	22 (15.1%)	0.749
Renal and ureteric stone^e^	26 (17.8%)	25 (17.1%)	1.000
Renal stone^e^	84 (57.5%)	85 (58.2%)	1.000
Staghorn stone^e^	13 (8.9%)	14 (9.6%)	1.000

^c^Normally distributed variable data analysed with paired-sample t test

^d^Nonnormally distributed variable data analysed with the Wilcoxon-Mann–Whitney test

^e^Categorical variable data analysed with the McNemar test

**Table 3:** Intraoperative conditions and early postoperative complications in the two groups

	Group A	Group B	*P* value
Operative time (min)^d^	79.5 ± 16.2	81.0 ± 14.5	0.184
Channel size^e^			
F18	82	79	0.724
F20	64	67	
Blood Leukocyte Counts (10^9^/L)^d^	9.1 ± 3.0	9.2 ± 2.9	0.949
Haemoglobin decrease (g/dl)^d^	10.0 ± 7.7	9.1 ± 6.7	0.390
VAS pain score			
Postoperative day 1^d^	2.18 ± 0.71	4.09 ± 1.35	< 0.001
Discharge day^d^	1.29 ± 0.72	2.31 ± 1.05	0.021
Clavien-Dindo classification			
Grade I			
Fever^e^	21 (14.4%)	23 (15.7%)	0.871
Analgesic requirement (%)^e^	19 (13.0%)	30 (20.5%)	0.005
Urinary extravasation (%)^e^	3 (2.1%)	9 (6.2%)	0.146
Grade II			
Sepsis (%)^e^	4 (2.7%)	3(2.1%)	1.000
Bleeding (%)^e^	12 (8.2%)	10 (6.8%)	0.617
F18^e^	7	5	0.594
F20^e^	5	5	1.000
Transfusion rate (%)^e^	3 (2.1%)	2 (1.4%)	1.000
Grade III			
Embolization (%)^e^	6 (4.1%)	2 (1.4%)	0.289
Residual stone (%)^e^	15 (10.3%)	12 (8.2%)	0.690
Catheterization time (d)^d^	3.3 ± 1.2	4.2 ± 1.1	< 0.001
Hospital stay (d)^d^	6.8 ± 1.6	7.7 ± 1.6	< 0.001

^c^Normally distributed variable data analysed with paired-sample t test

^d^Nonnormally distributed variable data analysed with the Wilcoxon-Mann–Whitney test

^e^Categorical variable data analysed with the McNemar test

## Discussion

This study compared clinical data on preoperative and intraoperative conditions, as well as early postoperative complications, between tubeless PCNL and standard PCNL patients. Firstly, using the PSM method, we obtained two sets of data with similar preoperative baseline data. Secondly, the two groups of patients had similar results in terms of intraoperative conditions and early postoperative complications. Finally, the VAS pain score in tubeless PCNL group was significantly lower than that in standard PCNL group, and the postoperative catheterization time and hospital stay of the tubeless PCNL group were lower than those of the standard PCNL group. This indicates that tubeless PCNL does not increase the incidence of postoperative complications compared to standard PCNL and is safe and effective, while simplifying the steps of PCNL.

Since 1997, it was first reported that tubeless PCNL has greater advantages than standard PCNL [12], but due to limitations in equipment and technology at that time, it has not been widely applied. With the maturity of equipment and surgical technology, tubeless PCNL has once again received attention. The forms of tubeless PCNL mainly include: (1) totally tubeless PCNL refers to postoperative surgery where neither the nephrostomy tube nor the ureteral stent is retained [13–15]; (2) partial tubeless PCNL, which means only retaining ureteral stents or double J tubes after surgery; (3) the modified tubeless PCNL, that is, only the indwelling ureteric stent was given after the operation, and it was pulled out after 1–2 days of observation without bleeding in hospital [16]. This study used partially tubeless PCNL and used PSM to reduce the difference in baseline data between the two groups of patients before surgery, resulting in more reliable results.

At present, the selection of patients in most studies on tubeless PCNL usually requires the following conditions [17]: (1) stone diameter < 3 cm; (2) single channel PCNL; (3) no residual stones, etc. However, there are also cases of staghorn calculi, complicated with renal insufficiency, abnormal renal anatomy, multi-channel, elderly patients, double kidney stone disease and other complex cases, all of which have successfully carried out tubeless PCNL [18–20]. There are also relevant studies that have optimized the method of tubeless PCNL [21]. After removing the plastic sheath, a safety guide wire is retained in the puncture channel, and the renal fistula opening is observed for 5–10 min. If there is no obvious bleeding at the nephrostomy opening, and there is no obvious damage to the renal pelvis and ureter, tubeless treatment can be performed. If you are concerned about arterial or massive venous bleeding, place a nephrostomy tube through a safe guide wire. This study mainly selected patients with non complex conditions for tubeless management after PCNL, and some particularly complex cases were not included. However, with the accumulation of cases and the enrichment of experience, it is also feasible for some patients with staghorn like stones, renal anatomical abnormalities, and multi-channel to undergo tubeless treatment.

There are many functions of indwelling a fistula tube after PCNL surgery, but the main function is to compress and stop bleeding [5]. Therefore, the most worrying complication of tubeless PCNL is postoperative bleeding. The main postoperative bleeding after tubeless PCNL is renal puncture channel bleeding, which may lead to bleeding into the renal collecting system and can be distinguished by observing the color changes of urine in the indwelling catheter. Another possibility is bleeding into the perirenal fascial space, which can be determined by observing vital signs, changes in hemoglobin, and ultrasound examinations. For severe bleeding, transcatheter angiography embolization has been identified as the preferred treatment method [22]. In this study, there was no significant increase in hemoglobin levels, incidence of bleeding, and cases treated with angiography embolization compared to standard PCNL after tubeless PCNL, indicating that tubeless PCNL has a higher safety in postoperative bleeding, consistent with literature reports [23]. The possible reasons why there is no increase in postoperative bleeding after tubeless PCNL include: (1) after the removal of the plastic sheath, the renal puncture channel can be contracted and closed, which is beneficial for reducing the incidence of renal bleeding. (2) the space within the perirenal fascia after suturing the nephrostomy opening is a relatively closed space. Even if there is a small amount of bleeding in the perirenal fascia, as the pressure increases, it may have a compressive hemostatic effect, and the bleeding has self-limiting properties. (3) If there is significant bleeding, there may be a huge hematoma in the perirenal fascia space, and the amount of bleeding is not self-limiting. This situation may involve renal artery rupture or arteriovenous fistula, and simply indwelling a nephrostomy tube cannot prevent renal bleeding, requiring transcatheter angiography embolization [24].

In terms of stone removal rate, there is no significant difference between tubeless PCNL and standard PCNL. Because the retention of a renal fistula tube is not related to the degree of stone removal. However, the stone clearance rates of the two groups were higher than those reported in previous literature, which may be due to different inclusion criteria for the selection of cases in this study. Due to the consideration that the stones have been completely cleared before undergoing tubeless treatment after PCNL, there is no need for secondary surgery, and the choice of tubeless method is related to the lower complexity of the stones in the patients included in this study. Totally tubeless PCNL may result in blood clots or large residual stones blocking the ureter, leading to poor renal urine drainage and symptoms such as urinary extravasation and low back pain. However, using partial tubeless PCNL and only retaining double J tubes can achieve unobstructed urine drainage, and the relief of urinary tract obstruction has a good effect on controlling postoperative infection. In this study, there were no significant differences in postoperative fever, sepsis, and postoperative related infection related indicators between the two groups. Related studies have shown that tubeless PCNL has been proven to be the safest and most effective compared to standard and totally tubeless PCNL [25]. Due to the absence of an indwelling nephrostomy tube, the VAS pain score in tubeless PCNL group was significantly lower than that in PCNL group [26]. It is also consistent with the results of this study. However, compared to the standard PCNL group, catheterization time and hospital stay in the tubeless PCNL group were shorter, which is similar to literature reports [24, 27]. It may be related to the process of omitting the clamping of the nephrostomy tube after the tubeless PCNL to observe the occurrence of symptoms such as fever, and then removing the nephrostomy tube.

Our current research has several limitations, including its retrospective nature and small sample size. The two groups of patients were not surgical patients at the same time period, and the inclusion criteria for the study subjects were limited, indicating that the tubeless treatment after PCNL is not universally applicable. In addition, it was not compared with totally tubeless PCNL. In addition, we will further design a large sample prospective randomized controlled study in the future, including long-term follow-up results to verify the safety of tubeless PCNL. At the same time, the inclusion criteria for tubeless treatment of PCNL require further stratified research.

## Conclusion

Tubeless PCNL is a simplification and improvement of the standard PCNL surgical process. According to the inclusion and exclusion criteria, it is safe and effective for selecting appropriate patients.

## Data Availability

The datasets used and/or analysed during the current study are available from the corresponding author on reasonable request.
